# The role of pre-pandemic depression for changes in depression, anxiety, and loneliness during the COVID-19 pandemic: Results from a longitudinal probability sample of adults from Germany

**DOI:** 10.1192/j.eurpsy.2022.2339

**Published:** 2022-11-03

**Authors:** Christoph Benke, Eva Asselmann, Theresa M. Entringer, Christiane A. Pané-Farré

**Affiliations:** 1 Department of Psychology, Clinical Psychology and Psychotherapy, Philipps University of Marburg, 35032 Marburg, Germany; 2 Differential and Personality Psychology, HMU Health and Medical University, 14471 Potsdam, Germany; 3 Socio-Economic Panel, German Institute for Economic Research (DIW Berlin), 10117 Berlin, Germany; 4 Center for Mind, Brain and Behavior (CMBB), University of Marburg and Justus Liebig University, Giessen, Germany

**Keywords:** Depressive disorder, lockdown measures, risk group, social distancing

## Abstract

**Background:**

The present study aims to delineate the role of preexisting depression for changes in common mental health problems during the COVID-19 pandemic.

**Methods:**

Using mixed-effects linear regression models, we analyzed data on the course of depressive (Patient Health Questionnaire-2) and anxiety (Generalized Anxiety Disorder-2) symptoms as well as loneliness (three-item UCLA Loneliness Scale) in a subset of the Socio-Economic Panel Study, a large and nationally representative household panel study from Germany. Participants were assessed during the first COVID-19 wave in Germany (March 31 to July 4, 2020; *n* = 6,694) and prospectively followed up at the peak of the second COVID-19 wave (January 18 to February 15, 2021; *n* = 6,038).

**Results:**

Overall, anxiety and depressive symptoms decreased, whereas loneliness increased from the first to the second COVID-19 wave. However, depressive symptoms increased and the surge in loneliness was steeper in those with versus without clinically relevant depressive symptoms in 2019 or a history of a depressive disorder before the COVID-19 pandemic. Anxiety symptoms remained stable throughout the pandemic in individuals with versus without clinically relevant depressive symptoms in 2019. Pre-pandemic depression was associated with overall higher depressive and anxiety symptoms and loneliness across both assessments. The stringency of lockdown measures did not affect the results.

**Conclusions:**

Our findings suggest that individuals with a history of depressive symptoms before the COVID-19 pandemic are at increased risk to experience an escalation of mental health problems due to the COVID-19 pandemic. Therefore, they might particularly profit from targeted prevention and early intervention programs.

## Introduction

Depressive symptomatology is prevalent among the general population and causes major psychological, social, and economic burdens [1, 2]. Individuals with increased depressive symptoms are at risk for several somatic sequelae (e.g., cardiovascular disease) and mental health issues including loneliness, anxiety, and the recurrence or deterioration of depression [3–5]. These mental health sequelae of depression, in turn, amplify the risk for somatic and mental disorder comorbidities, chronicity, as well as elevated psychosocial impairment and burden [1, 6–9]. Environmental adversities such as traumatic experiences, major stressful life events, and minor daily hassles are well known to play a central role in the development and course of depression and related mental health problems. Usually, they interact with other biological (e.g., genetic vulnerabilities), developmental (e.g., early childhood adversities), psychological (e.g., specific personality traits), and sociodemographic factors (e.g., gender) [10]. Moreover, an increasing body of evidence indicates that individuals affected by elevated depressive symptoms are more vulnerable to stressful events (i.e., higher stress reactivity and diminished recovery toward lower levels of stress; stress sensitization) [11] and may experience a greater number of depression-related stressors such as interpersonal problems or conflicts (stress generation) [10], which may cause a further increase in emotional disturbances in the long run.

This vulnerability to stressful events in depression may become particularly relevant in times in which individuals are faced with an inevitable stressor such as the COVID-19 pandemic that may threaten mental health. This assumption is corroborated by reports of increases in experienced distress, anxiety, depression, and loneliness from the time before the pandemic to the first wave of the pandemic in Germany [12, 13] and countries worldwide [14]. Recent studies on the longitudinal course of mental health during the COVID-19 pandemic revealed that anxiety, depression, and loneliness decreased during the easing of the first lockdown [15–19], but, again, increased while lockdown measures were extended and during the second and third COVID-19 waves in Europe [20, 21]. In contrast, there are studies demonstrating that mental health problems were relatively stable over time or even decreased during the COVID-19 pandemic [13, 22–24]. These inconsistent findings might be partly explained by differences in the composition of study populations and timing of the assessments.

It is particularly plausible to assume that especially individuals affected by elevated depressive symptoms before the pandemic would show unfavorable changes in mental health during the COVID-19 pandemic. Recent studies on the effect of depression on mental health during the COVID-19 pandemic, however, revealed inconclusive results. Current data collected during the initial phase of the COVID-19 pandemic indicated that previous or current depression was associated with higher depression, anxiety, loneliness, and COVID-19-related distress during the beginning of the COVID-19 pandemic [25, 26]. In contrast, individuals with versus without a pre-pandemic depression diagnosis did not differ in mental health changes during the initial COVID-19 phase [17, 27]. Beyond these findings from the initial COVID-19 phase, we know little about differences in the long-term course of depression, anxiety, and loneliness among individuals with versus without depressive symptoms or disorders prior to the pandemic so far. Most importantly, research on strictly prospective associations between depression levels prior to the pandemic and mental health changes during the pandemic is rare.

To close this research gap, we used data from the Socio-Economic Panel Study (SOEP), a nationally representative panel study from Germany. A subsample of SOEP participants (SOEP-CoV) was asked about depressive and anxiety symptoms as well as loneliness during the first COVID-19 wave (March to July 2020) and 6 months later, during the second COVID-19 wave (January to February 2021). In this publication, we aimed to test our *a priori* hypothesis of whether preexisting depressive symptomatology is associated with unfavorable changes in mental health during the COVID-19 pandemic. Therefore, we analyzed the role of depressive symptoms in 2019 and a history of a diagnosed depressive disorder (as derived from the regular SOEP waves before the pandemic) for changes in depressive and anxiety symptoms as well as loneliness during the COVID-19 pandemic (as derived from the additional SOEP COVID-19 assessments in 2020 and 2021). Elucidating the role of depression in mental health during an enduring stressful situation, such as the COVID-19 pandemic, may help to advance our understanding of the development and chronicity of depression and related mental health problems as well as inform public health and clinical approaches to prevention and intervention.

## Methods

### Participants

**Figure 1.**
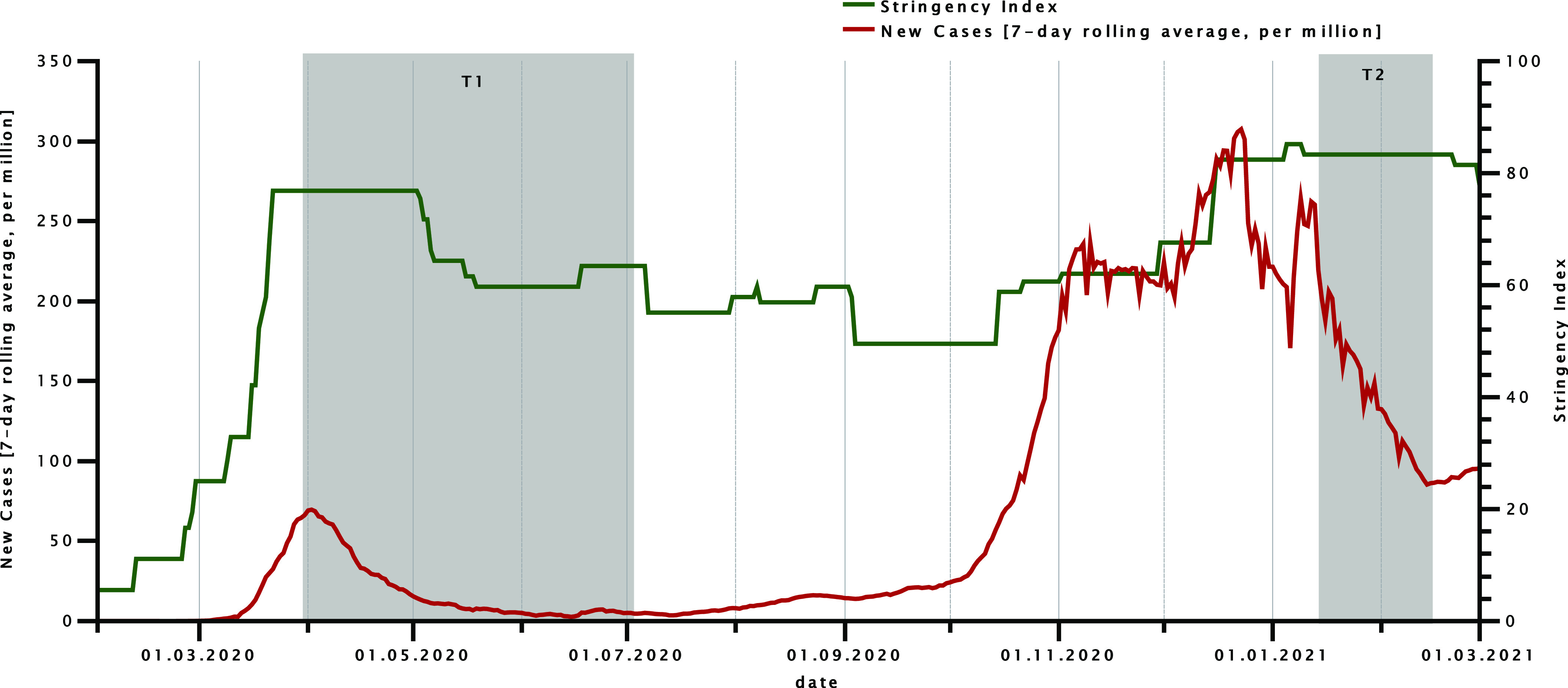
Course of new cases of COVID-19 (7-day rolling average, per million) and level of restrictions due to lockdown measures (indicated by the stringency index of the Oxford COVID-19 Government Response Tracker) during the COVID-19 pandemic in Germany (February 2020 to March 2021). The gray bars represent the time points and durations of the assessment phases (T1: first COVID-19 wave; T2: second COVID-19 wave).

We used data from a subset of SOEP, a nationally representative household panel with approximately 30,000 participants in 15,000 German households assessed since 1984 (v36) [28]. In 2020, a random SOEP subsample (SOEP-CoV) of 6,694 regular SOEP respondents was assessed during the first COVID-19 wave in Germany (March 31 to July 4, 2020) and, then, prospectively followed up at the peak of the second COVID-19 wave in Germany (*n* = 6,038), that is, from January 18 to February 15, 2021 [29]. As can be seen in Figure 1, the overall stringency of the government’s imposed lockdown measures was higher during the second versus first assessment period. All participants of the SOEP-CoV subsample were assessed using computer-assisted telephone interviews. The characteristics of the present study sample are summarized in Table 1. As shown in Table 1, the mean age of the sample was 54.2 years (age range: 18–99 years). In the present sample, 60.8% were female, and 32.6% had tertiary education. The mean equivalized household income was 2,237€ per month. Further information on the SOEP and SOEP-CoV is presented in the supplementary methods (see the Supplementary Material).

**Table 1.** Sample characteristics.

Age in years, *M* (*SD*)	54.2 (15.7)
Age groups, *N* (%)	
18–29	404 (6.72%)
30–49	1,929 (32.1%)
50–69	2,543 (42.3%)
>70	1,137 (18.9%)
Gender, *N* (%)	
Male	2,358 (39.2%)
Female	3,655 (60.8%)
Education (CASMIN classification), *N* (%)	
Primary	1,371 (23.9%)
Secondary	2,489 (43.5%)
Tertiary	1,867 (32.6%)
Occupation, *N* (%)	
Non-employed	2,154 (35.8%)
Employed	3,859 (64.2%)
Equivalized income in Euro (per month), *M* (*SD*)	2,237 (1322)
History of diagnosed depressive disorder, *N* (%)	
No depression	5,286 (87.9%)
Depression	727 (12.1%)

*Abbreviations*: CASMIN, Comparative Analysis of Social Mobility in Industrial Nations; M, mean; SD, standard deviation.

### Measures

#### Sociodemographic variables

Sociodemographic information, such as age, gender, and education, was assessed during the COVID-19 assessment or derived from previous assessment waves. Education was stratified as primary, secondary, and tertiary based on the highest level of education or vocational training according to the Comparative Analysis of Social Mobility in Industrial Nations. The equivalized household income included all disposable monthly household incomes, including all types of after-tax and transfer income. Differences in household size are considered by equivalization, that is, divided by the square root of the number of household members.

#### Outcome measures

*Loneliness.* In 2020 and 2021, loneliness during the past 2 weeks was assessed with the German version of the three-item UCLA Loneliness Scale [30] and rated on a four-point Likert scale (hardly, ever, some of the time, or often). Very similar to previous research [30], our study demonstrated acceptable internal consistency for the UCLA Loneliness Scale (i.e., α = 0.71).

*Depressive and anxiety symptoms.* In 2020 and 2021, depressive symptoms over the past 2 weeks were assessed with the two-item Patient Health Questionnaire-2 (PHQ-2; scores range from 0 to 6) and anxiety was assessed with the two-item Generalized Anxiety Disorder-2 (GAD-2; scores range from 0 to 6) questionnaire [31–33]. Similar to previous studies [31, 32], in the present sample, the internal consistency was α = 0.68 for the PHQ-2 and α = 0.68 for the GAD-2.

#### Predictors

*Depression diagnosis.* In 2019, individuals were asked to indicate whether a doctor had ever diagnosed a depressive disorder.

*Depressive symptoms in 2019.* In 2019, depressive symptoms were assessed using the PHQ-2. PHQ-2 scores of 3 and above indicate a depressive disorder with a sensitivity of 79% and a specificity of 86% [31].

*Restrictions due to public health measures in 2020.* In 2020, 14 forms of restriction measures that have been suggested to disrupt self-regulated and psychologically relevant behavior of individuals were systematically documented for each of the 16 German federal states on a day-by-day basis (e.g., prohibition to meet with others in public places, closure of kindergartens or daycare, and prohibition to leave the apartment without reason) by the Leibniz Institute for Psychology Information (ZPID, Germany; [51]). Each type of restriction was coded as not present (=0), partially (=1), or fully (=2) in place. A sum score of the level of restriction measures was computed per day to determine the daily level of personal and social restrictions resulting from public health measures in each federal state. Afterward, we matched the restriction scores of the respective federal state with the participants’ federal state. A higher sum score indicates a higher level of lockdown restrictions in the respective participants’ federal state on the day of the telephone interview. The stringency of lockdown measures significantly differed between federal states (see Supplementary Figure S1).

### Statistical analyses

All analyses were conducted using mixed-effects linear regression models with repeated measurement occasions (Level 1) nested within persons (Level 2), nested within federal states (Level 3). First, we analyzed changes in depressive and anxiety symptoms as well as loneliness from 2020 to 2021. Specifically, we regressed the score of the respective outcome (depressive symptoms, anxiety symptoms, or loneliness) on a timing variable (Level 1), coded with 0 at the first wave and coded with 1 at the second wave of the SOEP COVID-19 assessments. To investigate the role of (a) pre-pandemic depressive symptoms (PHQ-2 score in 2019) and (b) a history of a depressive disorder (no vs. yes) for changes in the respective outcome from 2020 to 2021, an interaction term between the respective predictor (i.e., depressive symptoms or disorder) and the timing variable was included in the respective model. Significant interactions were probed using simple slope analyses [34] to evaluate the significance of the time effects (i.e., change from 2020 to 2021) for conditional values of the moderator (for pre-pandemic depression diagnosis or clinically relevant depression assessed by the PHQ-2 in 2019: no vs. yes [0 vs. 1]). In the next step, we added the level of lockdown restrictions in 2020 and their interaction with pre-pandemic depressive symptoms or pre-pandemic depression diagnosis and time to the models. All analyses were controlled for age, gender, income, and education and conducted with R (version 4.1.2 [35]; packages: lme4, jtools, tidyverse, and ggplot). The alpha level was set to 0.05.

## Results

### Change in depression, anxiety, and loneliness from 2020 to 2021

As can be seen in Table 2, depressive and anxiety symptoms decreased over the course of the COVID-19 pandemic from 2020 to 2021 (depressive symptoms: *β* = −0.02, *p* = 0.020; anxiety symptoms: *β* = −0.03, *p* < 0.001; see Supplementary Table S1). Conversely, loneliness increased during the same period (*β* = 0.05, *p* < 0.001; see Supplementary Table S1).

**Table 2.** Means (SD) and percentages of individuals above the cutoff scores for depression and anxiety as well as loneliness in 2019 (before the pandemic), 2020 (first COVID-19 wave), and 2021 (second COVID-19 wave) in the overall sample and individuals with versus without a pre-pandemic depression diagnosis or clinically relevant depressive symptoms in 2019.

	Depressive symptoms		Anxiety symptoms		Loneliness	
	*M* (*SD*)	Cutoff ≥3	*M* (*SD*)	Cutoff ≥3	*M* (*SD*)	Cutoff ≥6
Overall						
2019	0.96 (1.24)	9.7%	0.74 (1.14)	6.8%	–	–
2020	1.28 (1.24)	13.9%	0.99 (1.14)	8.7%	5.05 (2.54)	39.5%
2021	1.24 (1.25)	12.0%	0.90 (1.13)	7.9%	5.32 (2.72)	43.9%
Depression diagnosis						
No						
2019	0.82 (1.10)	7.0%	0.60 (0.97)	4.3%	–	–
2020	1.21 (1.20)	12.6%	0.90 (1.07)	6.9%	4.99 (2.50)	38.5%
2021	1.14 (1.18)	10.1%	0.81 (1.04)	6.0%	5.22 (2.69)	42.5%
Yes						
2019	1.93 (1.69)	28.9%	1.71 (1.71)	24.5%	–	–
2020	1.82 (1.43)	23.6%	1.61 (1.44)	21.8%	5.48 (2.85)	46.7%
2021	1.93 (1.51)	25.8%	1.58 (1.45)	21.8%	5.99 (2.89)	54.0%
Clinically relevant depressive symptoms in 2019 [cut-off ≥ 3]						
No						
2019	0.65 (0.77)	–	0.54 (0.83)	2.8%	–	–
2020	1.21 (1.20)	12.5%	0.92 (1.08)	7.3%	4.98 (2.51)	38.1%
2021	1.13 (1.18)	9.8%	0.83 (1.06)	6.5%	5.23 (2.68)	42.6%
Yes						
2019	3.83 (1.06)	–	2.53 (1.89)	44.8%	–	–
2020	1.94 (1.44)	27.2%	1.57 (1.46)	21.5%	5.65 (2.79)	50.8%
2021	2.16 (1.54)	33.6%	1.60 (1.51)	21.9%	6.04 (3.06)	54.2%

*Note:* Depressive symptoms were assessed by PHQ-2, scores range from 0 to 6; anxiety symptoms were assessed by GAD-2, scores range from 0 to 6; loneliness was assessed by the three-item UCLA Loneliness Scale, scores range from 0 to 12. Loneliness was not assessed in 2019.

*Abbreviations*: GAD-2, Generalized Anxiety Disorder-2; M, mean; PHQ-2, Patient Health Questionnaire-2; SD, standard deviation.

### Effects of pre-pandemic depressive symptoms on changes in depression, anxiety, and loneliness

**Figure 2.**
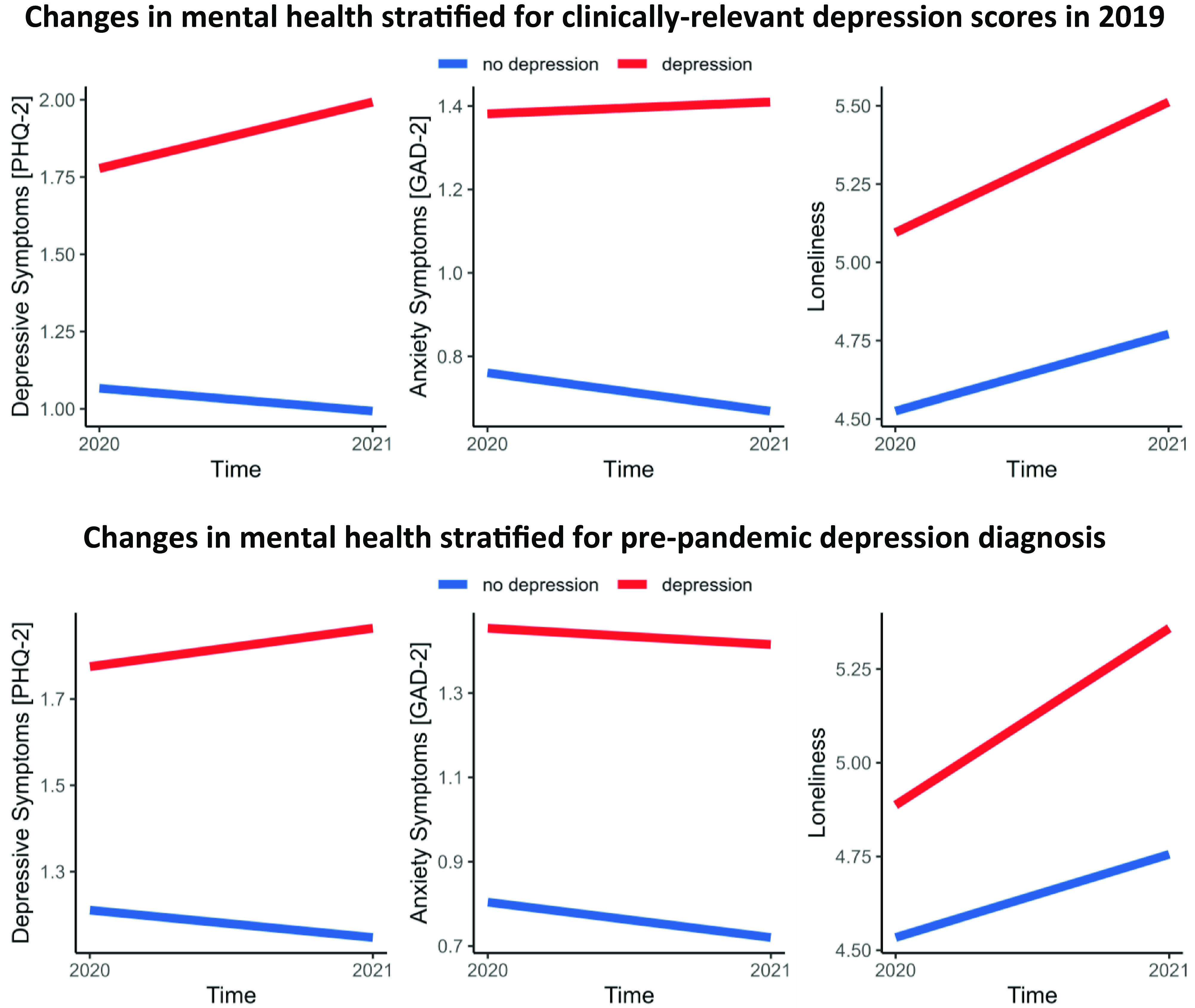
Course of depressive (PHQ-2) and anxiety symptoms (GAD-2) as well as loneliness (three-item UCLA Loneliness Scale) depending on the presence of a probable depressive disorder in 2019 (assessed by the PHQ-2; PHQ-2 scores of 3 and above indicate a probable depressive disorder; upper panel) or a diagnosed depressive disorder before the pandemic (lower panel). The lines represent conditional effects derived from simple slope analyses. *Abbreviations:* GAD-2, Generalized Anxiety Disorder-2; PHQ-2, Patient Health Questionnaire-2.

Pre-pandemic depressive symptoms were associated with overall higher levels of depressive and anxiety symptoms as well as loneliness in 2020 and 2021 (all *p* < 0.001; see Supplementary Table S2). Moreover, changes in depressive symptoms during the pandemic varied as a function of pre-pandemic depressive symptoms in 2019 (pre-pandemic depressive symptoms × time: *β* = 0.03, 95% CI [0.02; 0.05], *p* < 0.001). To assess the direction of interaction in greater detail, we distinguished between individuals above and below the clinical cutoff PHQ-2 score for a potential depression diagnosis in 2019. *Post hoc* simple slope analyses revealed that individuals above the cutoff score experienced an increase of depressive symptoms from 2020 to 2021 (*p* < 0.001), whereas individuals below the cutoff score experienced a decrease (see the upper-left panel of Figure 2; *p* < 0.001).

Moreover, higher levels of pre-pandemic depressive symptoms (PHQ-2 score in 2019) predicted a steeper increase in loneliness from 2020 to 2021 (pre-pandemic depressive symptoms × time: *β* = 0.02, 95% CI [0.01; 0.04], *p* = 0.001, all *post hoc* simple slope analyses *p* < 0.001; see the right panel of Figure 2).

Changes in anxiety symptoms did not vary significantly by pre-pandemic depressive symptoms (pre-pandemic depressive symptoms × time: *p* > 0.05). However, as depicted in the upper panel of Figure 2, anxiety symptoms did not change in individuals above the cutoff PHQ-2 score for a potential depression diagnosis in 2019 (*p* > 0.05), but decreased in individuals below the cutoff score (*p* < 0.001).

### Effect of a pre-pandemic depression diagnosis on the changes in depression, anxiety, and loneliness

A pre-pandemic depression diagnosis was associated with overall higher levels of depressive and anxiety symptoms as well as loneliness in 2020 and 2021 (all *p* < 0.001; see Supplementary Table S3). Most interestingly, depressive symptoms increased from 2020 to 2021 in individuals with (*p* = 0.03), but decreased in individuals without a pre-pandemic depression diagnosis (*p* < 0.001; pre-pandemic depression diagnosis × time: *β* = 0.06, 95% CI [0.02; 0.11], *p* = 0.009; see the lower panel of Figure 2). Similarly, the increase in loneliness from 2020 to 2021 was stronger in individuals with versus without a pre-pandemic depression diagnosis (pre-pandemic depression diagnosis × time: *β* = 0.05, 95% CI [0.01; 0.09], *p* = 0.028; all *post hoc* simple slope analyses *p* < 0.001). Change in anxiety during the pandemic did not differ significantly between individuals with versus without a pre-pandemic depression diagnosis (*p* > 0.05).

### Effects of pre-pandemic depressiveness and level of lockdown restrictions on the course of depression, anxiety, and loneliness

The stringency of lockdown restrictions in 2020 did not interact significantly with pre-pandemic levels of depressive symptoms or a pre-pandemic depression diagnosis in predicting changes in depression, anxiety, or loneliness from 2020 to 2021 (all *p* > 0.05; see supplementary Tables S4 and S5).

## Discussion

In the present study, we examined the effect of pre-pandemic depression on the course of depressive and anxiety symptoms as well as loneliness during the COVID-19 pandemic in a large probability sample of adults from Germany. We found that depressive and anxiety symptoms decreased, whereas loneliness increased from the first to the second wave of the COVID-19 pandemic in Germany. However, these symptom changes varied as a function of pre-pandemic depressive symptoms in 2019 and a diagnosed depressive disorder before the COVID-19 pandemic: depressive symptoms increased and the surge in loneliness was steeper in those with versus without clinically relevant depressive symptoms in 2019 or a history of a depressive disorder before the COVID-19 pandemic. Changes in anxiety symptoms did not vary as a function of a pre-pandemic depressive disorder. However, throughout the pandemic, anxiety symptoms remained stable in individuals with but declined in individuals without clinically relevant depressive symptoms prior to the pandemic. The stringency of lockdown measures did not affect the results.

Taken together, our findings suggest that the long-term course of depressive and anxiety symptoms as well as loneliness during the COVID-19 pandemic is less favorable in vulnerable individuals with versus without pre-pandemic depression. These results are in line with evidence that depression relates to less favorable responses to inevitable and uncontrollable/unpredictable stressors [36, 37]. However, they conflict with previous evidence that mental health problems during the *early* stage of the COVID-19 pandemic improved in individuals with and without pre-pandemic mental health problems [19, 27]. These findings might indicate that individuals with preexisting depression psychologically adapt to the demands of the pandemic in the short term, but show impaired adaptation and related exacerbation of mental health issues upon repeated or ongoing stressors.

Our finding that pre-pandemic depression predicted a greater increase of depression and loneliness during the pandemic is consistent with previous evidence that depression prospectively predicts higher loneliness and depressive symptoms [3, 38]. The potential underlying mechanisms of these associations are multifaceted. For example, depression not only relates to social withdrawal, but also interpersonal problems, interpersonal avoidance, and withdrawal of social support [10, 39, 40]. Such factors might amplify the adverse effect of COVID-19-related social distancing and isolation and explain our results. At the same time, feelings of loneliness might reinforce feelings of depression and vice versa [3, 5], leading to a vicious cycle of symptom escalation in particularly vulnerable individuals over time.

Particular strengths of this study are the prospective-longitudinal design, the inclusion of pre-pandemic data, and the use of a probability sample of a nationally representative household panel, which have been highly recommended as it reduces the risk of sampling and recall biases [41, 42]. In addition to these strengths, the following limitations need to be taken into account. Based on previous evidence, we assumed that the COVID-19 pandemic represents a stressor for most people. As the actual degree of experienced distress might have varied between individuals, future research ought to include appropriate measures to assess stress responses. Moreover, the present findings relied on self-report data and brief screening instruments such as the GAD-2. To obtain a more nuanced characterization of the course of anxiety, comprehensive assessments above and beyond generalized anxiety symptoms are needed. We did not assess information regarding a previous psychiatric or psychological treatment, which, indeed, should be addressed in future studies. Moreover, evidence from previous studies suggests that changes in mental health during the COVID-19 pandemic depend on the timing of the assessment (e.g., assessments during lockdown vs. easing of lockdown restrictions) [15–17, 19]. In the present study, the stringency of lockdown measures was slightly higher during the second compared with the first assessment. However, because only two assessment time points during the pandemic were considered in the present study, we were not able to reveal potential changes associated with a decrease in the stringency of lockdown measures.

## Conclusion

In the present study, individuals with elevated depressive symptoms or a history of a diagnosed depressive disorder were identified as a risk group for an unfavorable course of mental health problems during the COVID-19 pandemic. The observed persistence of anxiety symptoms as well as the increases in depressive symptoms and loneliness in this group might increase the risk for the clinical manifestation, exacerbation, or chronicity of depressive symptomatology and related somatic and mental health sequels. In particular, loneliness—a common concomitant feature of depression—has been associated with an increased risk for several mental disorders and somatic diseases in general [6, 8, 9, 43]. Thus, targeting loneliness in prevention and intervention in this particularly vulnerable group would indeed help to mitigate these sequelae, as already shown in previous randomized controlled trials [44, 45]. Moreover, addressing COVID-19-related worries or generalized worries in the context of online cognitive-behavioral interventions has also been proved to be effective in reducing worries but also depression and anxiety [46, 47]. Interestingly, recent advances in the field of online interventions indicate that mental health problems (e.g., restrictive eating, hopelessness, depression, and anxiety) can even be reduced via an online, self-guided single session intervention targeting behavioral activation or a growth mindset [48]. Moreover, previous studies found that younger individuals are more likely to experience loneliness as well as other mental health issues [49, 50]. Thus, a systematic evaluation of the impact of age in future studies might promote a more precise identification of at-risk persons. Finally, further research is needed to monitor potentially adverse long-term consequences in particularly vulnerable individuals with elevated depressive symptoms and to inform the health care system to implement evidence-based intervention strategies to mitigate potentially adverse consequences.

## Data Availability

The data can be accessed via the research data center of the SOEP.

## Supplementary material

For supplementary material accompanying this paper visit https://doi.org/10.1192/j.eurpsy.2022.2339. click here to view supplementary material
