# Supplementary material for: The role of pre-pandemic depression for changes in depression, anxiety, and loneliness during the COVID-19 pandemic: Results from a longitudinal probability sample of adults from Germany
Source: Eur Psychiatry. 2022 Nov 3;65(1):e76. doi: 10.1192/j.eurpsy.2022.2339 (PMC9706309; doi:10.1192/j.eurpsy.2022.2339)
Supplement: Supplementary file 1 [file S0924933822023392sup001.docx]

**Supplementary Materials**

**The role of pre-pandemic depression for changes in depression, anxiety, and loneliness during the COVID-19 pandemic:**

**results from a longitudinal probability sample of adults from Germany**

**Supplementary methods**

**Participants**

Information on the SOEP-COV dataset can be retrieved from www.soep-cov.de. Detailed information on the SOEP, including the sample structure, subsamples, and panel attrition, has been previously presented ([Goebel et al., 2019](#_ENREF_12); [Kroh et al., 2018](#_ENREF_23)) and can be found at <https://paneldata.org/soep-core>. All procedures and measures collected in the SOEP are described at <https://data.soep.de/soep-core>. The SOEP data are available for scientific use from the DIW Berlin after signing a contract on data distribution (<https://www.diw.de/en/diw_02.c.222829.en/access.html>). Because this study only involved secondary analyses of anonymized SOEP data provided by the DIW Berlin, additional ethical approval was not required. The authors assert that all procedures contributing to this work comply with the Helsinki Declaration of 1975, as revised in 2013.

**Measures**

***Restrictions due to public health measures in 2020***

**
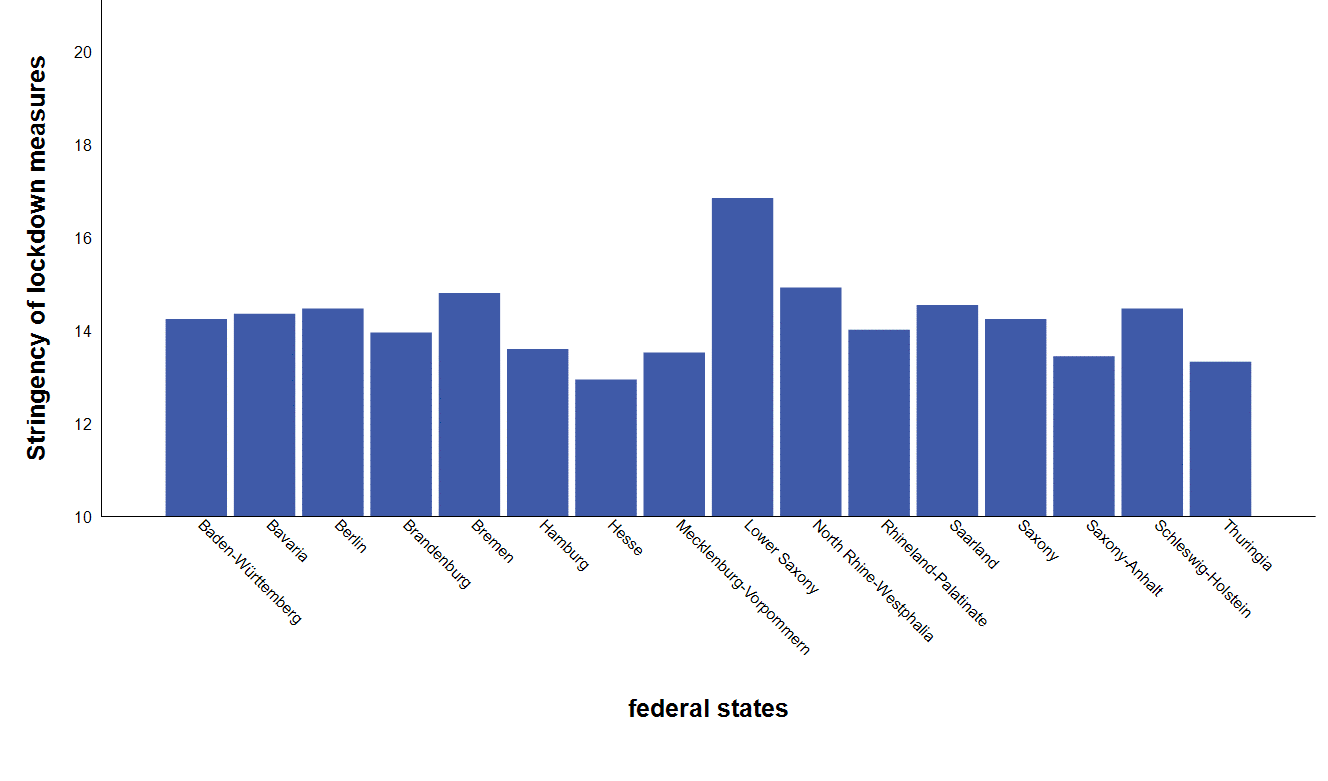
**

**Figure S1.** Average stringency of lockdown measures during the first COVID-19 wave for each German federal state based on data provided by the Leibniz Institute for Psychology Information (ZPID, Germany; Steinmetz et al., 2020). Stringency of lockdown measures significantly differ between federal states, *F*(15, 1469.99) = 21.95, *p* < .001.

**Supplementary results**

***Table S1.*** Results of mixed effects linear regression models examining the change in depressive and anxiety symptoms and loneliness from 2020 to 2021

|  | Depressive Symptoms | | | Anxiety Symptoms | | Loneliness | | | |
| --- | --- | --- | --- | --- | --- | --- | --- | --- | --- |
| Predictors | *Beta* | *95% CI* | *p* | *Beta* | *95% CI* | *p* | *Beta* | *95% CI* | *p* |
| intercept | -0.01 | -0.07 – 0.04 | **0.018** | -0.11 | -0.17 – -0.04 | **<0.001** | -0.19 | -0.26 – -0.12 | **<0.001** |
| age | -0.08 | -0.10 – -0.06 | **<0.001** | -0.10 | -0.13 – -0.08 | **<0.001** | -0.08 | -0.10 – -0.06 | **<0.001** |
| gender | 0.19 | 0.15 – 0.24 | **<0.001** | 0.25 | 0.20 – 0.29 | **<0.001** | 0.28 | 0.23 – 0.32 | **<0.001** |
| education - secondary | -0.13 | -0.19 – -0.08 | **<0.001** | -0.08 | -0.14 – -0.02 | **0.006** | 0.05 | -0.01 – 0.11 | 0.112 |
| education - tertiary | -0.15 | -0.21 – -0.09 | **<0.001** | -0.05 | -0.11 – 0.01 | 0.113 | -0.05 | -0.11 – 0.02 | 0.167 |
| income | -0.10 | -0.12 – -0.07 | **<0.001** | -0.08 | -0.10 – -0.05 | **<0.001** | -0.06 | -0.08 – -0.04 | **<0.001** |
| time | -0.02 | -0.03 – -0.00 | **0.020** | -0.03 | -0.05 – -0.02 | **<0.001** | 0.05 | 0.03 – 0.06 | **<0.001** |

*Note:* All variables were standardized according to Gelman (2008)

***Table S2.*** Results of mixed effects linear regression models examining the effect of pre-pandemic depressive symptoms (in 2019) on the change in depressive and anxiety symptoms and loneliness from 2020 to 2021

|  | Depressive Symptoms | | | Anxiety Symptoms | | | Loneliness | | |
| --- | --- | --- | --- | --- | --- | --- | --- | --- | --- |
| Predictors | *Beta* | *95% CI* | *p* | *Beta* | *95% CI* | *p* | *Beta* | *95% CI* | *p* |
| intercept | -0.02 | -0.07 – 0.03 | 0.402 | -0.11 | -0.17 – -0.06 | **<0.001** | -0.19 | -0.26 – -0.12 | **<0.001** |
| age | -0.07 | -0.09 – -0.04 | **<0.001** | -0.09 | -0.11 – -0.07 | **<0.001** | -0.07 | -0.10 – -0.05 | **<0.001** |
| gender | 0.16 | 0.12 – 0.20 | **<0.001** | 0.22 | 0.17 – 0.26 | **<0.001** | 0.26 | 0.22 – 0.31 | **<0.001** |
| education - secondary | -0.10 | -0.15 – -0.05 | **<0.001** | -0.05 | -0.11 – 0.00 | 0.065 | 0.06 | 0.00 – 0.12 | **0.043** |
| education - tertiary | -0.10 | -0.16 – -0.04 | **0.001** | -0.01 | -0.07 – 0.05 | 0.850 | -0.02 | -0.09 – 0.04 | 0.453 |
| income | -0.07 | -0.09 – -0.05 | **<0.001** | -0.06 | -0.08 – -0.03 | **<0.001** | -0.05 | -0.07 – -0.03 | **<0.001** |
| time | -0.02 | -0.03 – -0.00 | **0.016** | -0.03 | -0.05 – -0.02 | **<0.001** | 0.05 | 0.03 – 0.06 | **<0.001** |
| depressiveness [PHQ-2] in 2019 | 0.29 | 0.27 – 0.31 | **<0.001** | 0.26 | 0.24 – 0.28 | **<0.001** | 0.12 | 0.09 – 0.14 | **<0.001** |
| time x depressiveness | 0.03 | 0.02 – 0.05 | **<0.001** | 0.01 | -0.00 – 0.02 | 0.196 | 0.02 | 0.01 – 0.04 | **0.001** |

*Note:* All variables were standardized according to Gelman (2008)

***Table S3.*** Results of mixed effects linear regression models examining the effect of a pre-existing depression diagnosis on the change in depressive and anxiety symptoms and loneliness from 2020 to 2021

|  | Depressive Symptoms | | | Anxiety Symptoms | | | Loneliness | | |
| --- | --- | --- | --- | --- | --- | --- | --- | --- | --- |
| Predictors | *Beta* | *95% CI* | *p* | *Beta* | *95% CI* | *p* | *Beta* | *95% CI* | *p* |
| intercept | -0.06 | -0.12 – -0.01 | **0.028** | -0.16 | -0.21 – -0.11 | **<0.001** | -0.20 | -0.27 – -0.14 | **<0.001** |
| age | -0.08 | -0.10 – -0.06 | **<0.001** | -0.10 | -0.12 – -0.08 | **<0.001** | -0.08 | -0.10 – -0.06 | **<0.001** |
| gender | 0.17 | 0.12 – 0.21 | **<0.001** | 0.22 | 0.17 – 0.26 | **<0.001** | 0.27 | 0.22 – 0.31 | **<0.001** |
| education - secondary | -0.13 | -0.19 – -0.08 | **<0.001** | -0.08 | -0.14 – -0.03 | **0.004** | 0.05 | -0.01 – 0.11 | 0.116 |
| education - tertiary | -0.14 | -0.20 – -0.08 | **<0.001** | -0.04 | -0.10 – 0.03 | 0.263 | -0.04 | -0.11 – 0.02 | 0.205 |
| income | -0.09 | -0.11 – -0.06 | **<0.001** | -0.06 | -0.09 – -0.04 | **<0.001** | -0.06 | -0.08 – -0.03 | **<0.001** |
| time | -0.03 | -0.04 – -0.01 | **0.002** | -0.04 | -0.05 – -0.02 | **<0.001** | 0.04 | 0.03 – 0.06 | **<0.001** |
| depression diagnosis | 0.51 | 0.45 – 0.58 | **<0.001** | 0.59 | 0.53 – 0.66 | **<0.001** | 0.18 | 0.11 – 0.25 | **<0.001** |
| time x depression diagnosis | 0.06 | 0.02 – 0.11 | **0.009** | 0.02 | -0.02 – 0.06 | 0.366 | 0.05 | 0.01 – 0.09 | **0.028** |

*Note:* All variables were standardized according to Gelman (2008)

***Table S4.*** Results of mixed effects linear regression models examining the effect of a pre-existing depression diagnosis and the stringency of lockdown measures on the change in depressive and anxiety symptoms and loneliness from 2020 to 2021

|  | Depressive Symptoms | | | Anxiety Symptoms | | | Loneliness | | |
| --- | --- | --- | --- | --- | --- | --- | --- | --- | --- |
| Predictors | *Beta* | *95% CI* | *p* | *Beta* | *95% CI* | *p* | *Beta* | *95% CI* | *p* |
| intercept | -0.06 | -0.12 – -0.01 | **0.030** | -0.17 | -0.23 – -0.10 | **<0.001** | -0.20 | -0.27 – -0.13 | **<0.001** |
| age | -0.08 | -0.10 – -0.06 | **<0.001** | -0.10 | -0.12 – -0.08 | **<0.001** | -0.08 | -0.11 – -0.06 | **<0.001** |
| gender | 0.17 | 0.12 – 0.21 | **<0.001** | 0.22 | 0.18 – 0.26 | **<0.001** | 0.27 | 0.22 – 0.31 | **<0.001** |
| education - secondary | -0.13 | -0.19 – -0.08 | **<0.001** | -0.08 | -0.14 – -0.03 | **0.004** | 0.04 | -0.01 – 0.10 | 0.137 |
| education - tertiary | -0.14 | -0.20 – -0.08 | **<0.001** | -0.04 | -0.10 – 0.02 | 0.212 | -0.04 | -0.10 – 0.02 | 0.208 |
| income | -0.09 | -0.11 – -0.06 | **<0.001** | -0.06 | -0.09 – -0.04 | **<0.001** | -0.06 | -0.08 – -0.03 | **<0.001** |
| time | -0.03 | -0.04 – -0.01 | **0.002** | -0.04 | -0.05 – -0.02 | **<0.001** | 0.04 | 0.03 – 0.06 | **<0.001** |
| depression diagnosis | 0.51 | 0.45 – 0.58 | **<0.001** | 0.59 | 0.52 – 0.65 | **<0.001** | 0.19 | 0.12 – 0.25 | **<0.001** |
| stringency of lockdown measures | 0.01 | -0.02 – 0.03 | 0.613 | -0.01 | -0.03 – 0.02 | 0.597 | 0.06 | 0.04 – 0.08 | **<0.001** |
| time x depression diagnosis | 0.06 | 0.01 – 0.11 | **0.010** | 0.02 | -0.02 – 0.06 | 0.367 | 0.04 | 0.00 – 0.09 | **0.045** |
| time x stringency | -0.03 | -0.04 – -0.01 | **0.002** | -0.00 | -0.02 – 0.01 | 0.840 | -0.05 | -0.06 – -0.03 | **<0.001** |
| depression diagnosis x stringency | -0.02 | -0.08 – 0.05 | 0.625 | -0.03 | -0.10 – 0.03 | 0.316 | 0.01 | -0.06 – 0.08 | 0.794 |
| time x depression diagnosis x stringency | 0.01 | -0.03 – 0.06 | 0.560 | 0.00 | -0.04 – 0.05 | 0.922 | -0.02 | -0.06 – 0.02 | 0.373 |

*Note:* All variables were standardized according to Gelman (2008)

***Table S5.*** Results of mixed effects linear regression models examining the effect of pre-pandemic depressive symptoms (in 2019) and the stringency of lockdown measures on the change in depressive and anxiety symptoms and loneliness from 2020 to 2021

|  | Depressive Symptoms | | | | | | Anxiety Symptoms | | | Loneliness | | |
| --- | --- | --- | --- | --- | --- | --- | --- | --- | --- | --- | --- | --- |
| Predictors | *Beta* | *95% CI* | | *p* | | *Beta* | *95% CI* | *p* | *Beta* | | *95% CI* | *p* |
| intercept | -0.02 | -0.07 – 0.03 | | 0.405 | | -0.11 | -0.17 – -0.06 | **<0.001** | -0.18 | | -0.25 – -0.12 | **<0.001** |
| age | -0.07 | -0.09 – -0.04 | | **<0.001** | | -0.09 | -0.11 – -0.07 | **<0.001** | -0.08 | | -0.10 – -0.05 | **<0.001** |
| gender | 0.16 | 0.12 – 0.20 | | **<0.001** | | 0.22 | 0.17 – 0.26 | **<0.001** | 0.26 | | 0.22 – 0.31 | **<0.001** |
| education - secondary | -0.10 | -0.15 – -0.05 | | **<0.001** | | -0.05 | -0.11 – 0.00 | 0.068 | 0.06 | | -0.00 – 0.12 | 0.051 |
| education - tertiary | -0.10 | -0.16 – -0.04 | | **0.001** | | -0.01 | -0.07 – 0.05 | 0.846 | -0.02 | | -0.09 – 0.04 | 0.450 |
| income | -0.07 | -0.09 – -0.05 | | **<0.001** | | -0.06 | -0.08 – -0.03 | **<0.001** | -0.05 | | -0.07 – -0.03 | **<0.001** |
| time | -0.02 | -0.03 – -0.00 | | **0.017** | | -0.03 | -0.05 – -0.02 | **<0.001** | 0.05 | | 0.03 – 0.06 | **<0.001** |
| depressiveness in 2019 | 0.29 | 0.27 – 0.31 | | **<0.001** | | 0.26 | 0.24 – 0.28 | **<0.001** | 0.12 | | 0.10 – 0.14 | **<0.001** |
| stringency of lockdown measures | 0.00 | -0.02 – 0.02 | | 0.841 | | -0.01 | -0.03 – 0.01 | 0.295 | 0.06 | | 0.04 – 0.08 | **<0.001** |
| time x depressiveness | 0.03 | 0.02 – 0.05 | | **<0.001** | | 0.01 | -0.00 – 0.02 | 0.195 | 0.02 | | 0.01 – 0.04 | **0.002** |
| time x stringency | -0.02 | -0.04 – -0.01 | | **0.002** | | -0.00 | -0.02 – 0.01 | 0.868 | -0.05 | | -0.06 – -0.03 | **<0.001** |
| depressiveness x stringency | -0.01 | -0.03 – 0.01 | | 0.368 | | 0.00 | -0.02 – 0.02 | 0.827 | 0.01 | | -0.01 – 0.03 | 0.435 |
| time x depressiveness x stringency | 0.01 | -0.01 – 0.02 | | 0.258 | | 0.00 | -0.01 – 0.02 | 0.661 | -0.00 | | -0.02 – 0.01 | 0.783 |
|  |  | |  | |  |  |  |  |  |  |  |  |

*Note:* All variables were standardized according to Gelman (2008)
